# Involvement of endocrine system in a patient affected by Glycogen storage disease 1b: speculation on the role of autoimmunity

**DOI:** 10.1186/1824-7288-40-30

**Published:** 2014-03-19

**Authors:** Daniela Melis, Roberto Della Casa, Francesca Balivo, Giorgia Minopoli, Alessandro Rossi, Mariacarolina Salerno, Generoso Andria, Giancarlo Parenti

**Affiliations:** 1 Department of Translational Medical Sciences, Section of Pediatrics, Federico II University, Via S. Pansini 5, 80131 Naples, Italy

**Keywords:** Glycogen storage disease 1b, Autoimmunity, Thyroiditis, Growth hormone deficiency, Inflammatory bowel disease

## Abstract

Glycogen storage disease type 1b (GSD1b) is an inherited metabolic defect of glycogenolysis and gluconeogenesis due to mutations of the SLC37A4 gene and to defective transport of glucose-6-phosphate. The clinical presentation of GSD1b is characterized by hepatomegaly, failure to thrive, fasting hypoglycemia, and dyslipidemia. Patients affected by GSD1b also show neutropenia and/or neutrophil dysfunction that cause increased susceptibility to recurrent bacterial infections. GSD1b patients are also at risk for inflammatory bowel disease. Occasional reports suggesting an increased risk of autoimmune disorders in GSD1b patients, have been published. These complications affect the clinical outcome of the patients. Here we describe the occurrence of autoimmune endocrine disorders including thyroiditis and growth hormone deficiency, in a patient affected by GSD1b. This case further supports the association between GSD1b and autoimmune diseases.

## Background

Glycogen storage disease type 1b (GSD1b; OMIM 232220) is an inherited metabolic defect due to mutations of the *SLC37A4* gene that encodes a microsomal glucose-6-phosphate transporter (G6PT) [1]. Decreased transport of glucose-6-phosphate (G6P) across the microsomal membrane causes inability of G6P to come in contact with the catalytic unit of glucose-6-phosphatase (G6Pase-alpha), insufficient conversion of G6P into glucose and phosphate, and defective glycogenolysis and gluconeogenesis [2,3]. The biochemical defect of GSD1b results in a phenotype characterized by hepatomegaly and failure to thrive, which is similar to that of other liver glycogenoses, and in typical biochemical abnormalities (fasting hypoglycemia, hyperlacticacidemia, hyperlipidemia, hyperuricemia). The treatment of GSD1b is mainly based on a dietary regimen with frequent meals, nocturnal gastric drip feeding and use of uncooked starches with low glycemic index.

In addition to the classical phenotype of glycogenoses, GSD1b is associated with neutropenia and/or neutrophil dysfunction that cause increased susceptibility to recurrent bacterial infections, aphthous stomatitis, and inflammatory bowel disease (IBD) [4,5].

Although these manifestations are highly debilitating and impact significantly on patients’ quality of life, their pathophysiology is still poorly characterized. Granulocyte-Colony Stimulating factor (G-CSF) is commonly used, in addition to the standard dietary treatment, for the management of neutrophil abnormalities in GSD1b patients and is effective in decreasing the frequency of infections [1,4-7].

These abnormalities of neutrophil count and function have been claimed to be a predisposing factor to autoimmune diseases [8,9]. Reports have been published in the recent literature on the association between GSD1b and IBD [4], autoimmune thyroiditis [10], growth hormone (GH) deficiency [11], and autoimmune myastenia gravis [12]. The possible role of autoimmunity in GH deficiency has also been reported [13].

Here we describe a patient affected by GSD1b that showed several manifestations of autoimmunity including IBD, autoimmune thyroiditis and autoimmune GH deficiency.

## Case presentation

GDN, a male, was the first child of third cousin parents. He was delivered by caesarean section. In the neonatal period he presented with hypoglycemia, seizures, hyperbilirubinemia (16 mg/dL), and hemolytic disease due to AB0 incompatibility.

At the age of three months he was first referred to Genetic Clinic Unit because of fasting hypoglycemia with seizures, liver enlargement, recurrent infections, and candidiasis. The diagnosis of GSD1b was based on the clinical presentation, on the typical biochemical profile with lactic acidosis, dyslipidemia, and hyperuricemia, and on the assay of glucose-6-phosphatase in fresh and frozen liver samples. When the molecular diagnosis of the disease became available, the diagnosis was confirmed by the mutational analysis of the *SLC37A4* gene, showing homozygosity for the mutation c.1211-1212delCT.

A diet based on frequent meals and nocturnal gastric drip-feeding was started and the patient was included in a follow-up program at our Department. Biochemical and growth parameters improved after the start of dietary treatment.

During the follow-up, however, GDN presented with many of the known complications of GSD1b.

At the age of 6 months neutropenia was detected. The median neutrophil count, over a 5-year follow-up period, was 1160/mm^3^, ranging between 230 and 4523. A therapy with G-CSF was started at the age of 6 months with a dose of 3–5 μg/kg/day.

Recurrent respiratory and gastro-intestinal infections and splenomegaly were also recorded.

When he was 8 years old, glomerular hyperfiltration and microalbuminuria were detected on routine biochemical evaluations and treatment with ACE-inhibitors was started to prevent further progression of renal disease.

In addition to the known complications of GSD1b, the patient presented with disorders suggesting autoimmunity.

At the age of 7 years, given the presence of stomatitis aphthosa and perianal ulcers, an ileocolonoscopy was performed showing signs of non-specific chronic inflammation. A scintigraphy with ^99m^Technetium-labeled autologous white cell (Tc-WCS) showed moderate to high degree neutrophil chemiotaxis at the ileocaecal junction. A terminal ileum ultrasonography showed increased bowel wall thickness. The treatment of the intestinal involvement was based on the use of an elemental diet and periodic administration of antibiotics.

Biochemical evidence of autoimmune thyroiditis with subclinical hypothyroidism was observed at the age of 12 years. The diagnosis was based on repeated increased basal TSH levels and thyroid auto-antibodies (Table 1), with abnormal echogenicity of the parenchyma on a thyroid ultrasound scan. During the follow-up, TSH levels were always below 10 μU/ml and thus he never started a replacement therapy with L-thyroxine [14,15]. At that time, a complete panel of serum autoantibodies was assayed showing increased antibodies against ANA, Anti-C1Q, Anti-C3d (Table 1).

**Table 1:** Endocrine profile at 14 years

			**Results**	**Reference values**
Thyroid	TSH (μU/ml)		5.6	0.5–4.5
	FT3 (pg/ml)		3.5	1.6–3.4
	FT4 (ng/dl)		1.2	0.7–1.7
	Anti-TPO (UI/ml)		57	0–40
	Anti-thyreoglobulin (UI/ml)		121	0–40
Growth hormone & pituitary	Basal GH (ug/l)		0.04	0.2–5
	IGF-I (ug/l)		53.9	100–500
	Anti-pituitary antibodies		1:8	0
Adrenals	Basal ACTH (ng/ml)		17.6	10–130
	Basal cortisol (ng/ml)		120	50–200
	ACTH test			
	Cortisol (ng/ml)	T0	120	50–200
		T30	236	>100
		T60	249	>100
		T90	260	>100
	Anti-adrenal gland antibodies		Absent	Absent
	Anti-ACTH antibodies		Absent	Absent
Auto-antibody profile	ANA (U/ml)		3.1	0–1
	Ds-DNA (UI/ml)		0	0–5
	ANCA (U/ml)		0	0–5
	ASCA (U/ml)		0	<20
	Cardiolipin (PLU/ml)		0	<10
	Phospholipid (U/ml)		0	<10
	Acetylcholine receptor (nmol/l)		0	0–0.25
	Anti-C1Q (U/ml)		140	1–40
	Anti-C3d (U/ml)		120	0–24

**Figure 1:**
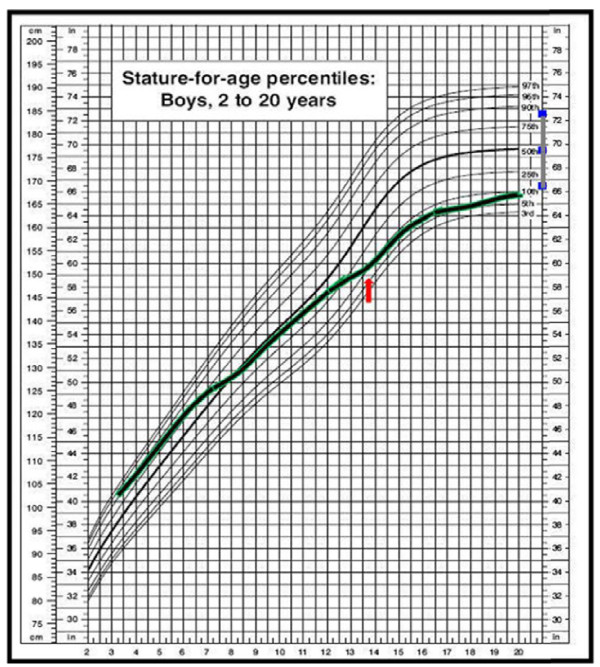
**Patient’s growth pattern.** Height velocity deceleration was observed when the patient was 15 years old. The arrow indicates the time GH-RH plus arginine dynamic test was performed. Final height was below mid-parental height.

**Figure 2:**
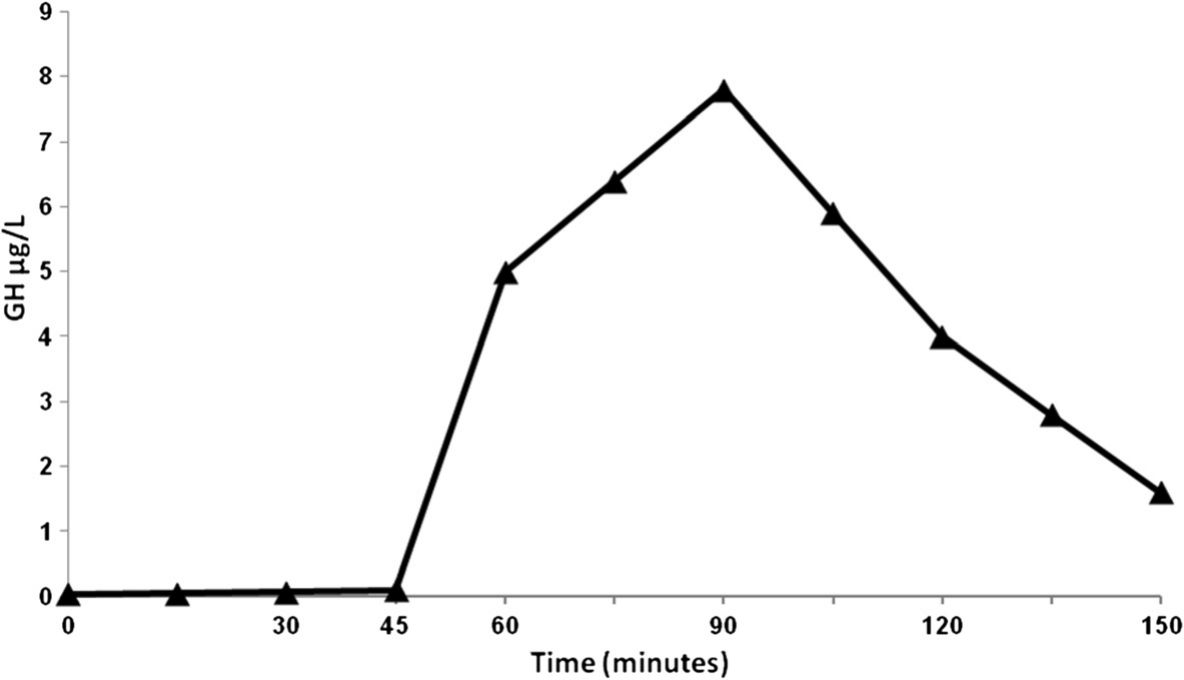
**Graphic representation of GH-RH plus arginine dynamic test demonstrating GH deficiency in the patient.** The patient is represented by ▲.

When he was 14-years-old, reduced height velocity (Figure 1), short stature, and retarded bone maturation (corresponding to 12 years) were observed. At that time pubertal stage was G2P2, TSH was 5.6 μU/ml with normal fT4 1.2 ng/dl (normal values 0.7-1.7). A GH-RH plus arginine test was performed, showing reduced GH response (peak 7,8 ng/dl) (Figure 2). High titer of anti-pituitary antibodies recognizing growth hormone-producing cells (>1:8) were also detected, indicating an autoimmune growth hormone deficiency. A pituitary Magnetic Resonance Imaging was normal, showing a normal gland architecture. The adrenal gland function was also evaluated, showing normal basal hormone levels and normal response to a dynamic low dose ACTH test (Table 1).

At the age of 16 years he also showed delayed pubertal development; also in this case no treatment was started. At that time, considering the increased number of infections and their seriousness and the appearance of skin pigmentation, adrenal function was re-evaluated, showing basal ACTH 15 ng/ml and basal cortisol 54 ng/ml. A dynamic low dose ACTH test was also performed showing a significant increase in cortisol levels (cortisol T0 51.1 ng/ml, T30 221 ng/ml, T60 229 ng/ml, T90 260 ng/ml), thus suggesting a normal functioning of the adrenal gland and only a slight pituitary dysfunction.

## Conclusions

We report on a patient presenting with different manifestations related to altered immune response, such as increased susceptibility to bacterial infections, autoimmune thyroiditis and autoimmune GH deficiency. In GSD1b, neutropenia and impaired neutrophil function represent the *primum movens* of the reduced response to infection.

The occurrence of autoimmune disorders has been reported in GSD1b only in the recent years and little is known about its pathophysiology. Different hypotheses may be considered to explain the presence of autoimmune disorders in GSD1b patients.

Firstly it is known that autoimmunity may be related to the defective function of neutrophils. In fact, there are several disorders in which abnormal function of neutrophil is associated with autoimmunity. For example carrier mothers of children with chronic granulomatous disease, an X-linked defect of phagocytosis, often develop discoid lupus [8]. Similarly, combined cellular and antibody deficiencies, such as Wiskott-Aldrich syndrome, are associated with an increased risk for juvenile rheumatoid arthritis and autoimmune hemolytic anemia [9].

Secondly, immunologic defects may determine a failure to exclude microbial antigens, thus resulting in chronic immunologic activation, with the rising of autoimmune manifestations [16]. In GSD1b patients, recurrent infections may lead to increased expression of MHC antigens or trigger the immune response through molecular mimicry and cross-reactivity between endogenous and microbial peptides.

In addition, it might be hypothesized that, in GSD1b patients, failure of neutrophils to achieve intracellular killing of bacteria may result in induction of lymphocyte T_H_l-type proliferation as a compensatory mechanism [16].

In our patient we observed only minor endocrine autoimmune manifestations, involving pituitary gland, thyroid and adrenal gland. Considering that TSH levels were always below 10 μU/ml and basal ACTH and cortisol were in the low reference range, he never started a specific therapy. As far as GH deficiency is concerned no replacement therapy was started, considering that rhGH is known to stimulate gluconeogenesis and to increase fasting insulin levels and insulin-resistance [17,18] thus interfering with glucose metabolism and possibly worsening metabolic imbalance in GSD1. In addition it is known the promoting effect of GH on the growth of liver cell adenomas [19], a known long-term complication in GSD1. Therefore, we decided to monitor auxological parameters before beginning therapy with rhGH, considering its potential dangerousness.

Although no hormone replacement therapy was started in our patient, it is difficult to predict whether in other patients these manifestations may contribute to the clinical picture of the diseases and impact on patients’ quality of life. It has been recently reported that patients with GSD1b are more likely to experience a poor quality of life [20].

In conclusion, given the known association between immunodeficiency and autoimmunity, we suggest monitoring GSD1b patients for autoimmune disorders.

## Consent

Written informed consent was obtained from the mother of the patient for the publication of this Case report. A copy of the written consent is available for being reviewed by the Editor-in-Chief of this journal.

## Abbreviations

GSD1b: Glycogen storage disease type 1b; G6P: Glucose-6-phosphate; G6Pase: Glucose-6-phosphatase; IBD: Inflammatory bowel disease; G-CSF: Granulocyte-Colony Stimulating factor; GH: Growth Hormone; Tc-WCS: Scintigraphy with ^99m^Technetium-labeled autologous white cell.

## Competing interests

The authors declare that they have no competing interests.

## Authors’ contributions

DM, RDC, FB, AR, GM, MCS followed up the patient over the 15-year follow-up, GP and GA were the consultant metabolic physicians, DM and GP wrote the paper. All authors read and approved the final manuscript.
